# Environmental Enrichment: Disentangling the Influence of Novelty, Social, and Physical Activity on Cerebral Amyloid Angiopathy in a Transgenic Mouse Model

**DOI:** 10.3390/ijms21030843

**Published:** 2020-01-28

**Authors:** Lisa S. Robison, Nikita Francis, Dominique L. Popescu, Maria E. Anderson, Joshua Hatfield, Feng Xu, Brenda J. Anderson, William E. Van Nostrand, John K. Robinson

**Affiliations:** 1 Department of Psychology, Stony Brook University, 100 Nicolls Road, Stony Brook, NY 11794, USA; robisol@amc.edu (L.S.R.); nikita_francis@uri.edu (N.F.); dominique_popescu@brown.edu (D.L.P.); mariaeanderson@gmail.com (M.E.A.); brenda.anderson@stonybrook.edu (B.J.A.); 2 Department of Neuroscience and Experimental Therapeutics, Albany Medical College, 47 New Scotland Ave, Albany, NY 12208, USA; 3 George & Anne Ryan Institute for Neuroscience, 130 Flagg Road, University of Rhode Island, Kingston, RI 02881, USA; jhatfield5210@gmail.com (J.H.); feng_xu@uri.edu (F.X.); wvannostrand@uri.edu (W.E.V.N.); 4 Department of Psychiatry and Human Behavior, Warren Alpert Medical School of Brown University, 700 Butler Drive, Providence, RI 02906, USA; 5 Department of Psychology, Farmingdale State College, 2350 Broadhollow Rd, Farmingdale, NY 11735, USA; 6 Department of Biomedical and Pharmaceutical Sciences, University of Rhode Island, Kingston, RI 02881, USA; 7 Department of Psychology, University of Rhode Island, Kingston, RI 02881, USA

**Keywords:** cerebral amyloid angiopathy, Alzheimer’s disease, enriched environment, exercise, reserve, resilience

## Abstract

Cerebral amyloid angiopathy (CAA) is the deposition of amyloid protein in the cerebral vasculature, a common feature in both aging and Alzheimer’s disease (AD). However, the effects of environmental factors, particularly cognitive stimulation, social stimulation, and physical activity, on CAA pathology are poorly understood. These factors, delivered in the form of the environmental enrichment (EE) paradigm in rodents, have been shown to have beneficial effects on the brain and behavior in healthy aging and AD models. However, the relative importance of these subcomponents on CAA pathology has not been investigated. Therefore, we assessed the effects of EE, social enrichment (SOC), and cognitive enrichment (COG) compared to a control group that was single housed without enrichment (SIN) from 4 to 8 months of age in wild-type mice (WT) and Tg-SwDI mice, a transgenic mouse model of CAA that exhibits cognitive/behavioral deficits. The results show that individual facets of enrichment can affect an animal model of CAA, though the SOC and combined EE conditions are generally the most effective at producing physiological, cognitive/behavioral, and neuropathological changes, adding to a growing literature supporting the benefits of lifestyle interventions.

## 1. Introduction

Cerebral amyloid angiopathy (CAA), the deposition of amyloid protein in the cerebral vasculature, is common in the aging population and often co-exists with Alzheimer’s disease (AD) [1]. CAA is associated with several other small vessel disease pathologies, including cerebral hemorrhages, white matter lesions, cortical microinfarcts, and perivascular inflammation [2,3,4,5,6,7]. There is a strong positive correlation between CAA and AD symptoms and pathology [8]. CAA is specifically linked to deficits in several cognitive domains, including perceptual slowing and episodic memory [9], and has been shown to be a better predictor of cognitive decline than parenchymal Aβ deposition in mouse models [10]. Unfortunately, there is currently no efficacious pharmaceutical intervention for CAA. Therefore, research to identify modifiable lifestyle factors that may reduce the risk and/or progression of dementia has come to the forefront. Epidemiological studies have identified a number of factors associated with a reduced risk of cognitive decline, including being physically active [11,12,13,14] and cognitive and social stimulation [11,15,16,17,18]. To help elucidate the causal relationship between these factors and outcomes, animal models can provide the opportunity to determine the contribution of these lifestyle factors to differential outcomes in pathology, cognitive performance, and additional related behaviors.

The complex interaction between physical-, social-, and cognitive-stimulating components can be modeled in rodents using the enriched environment (EE) paradigm. In the EE, rodents are usually group housed in a large cage with an exercise wheel, toys, and tunnels of varying shapes, colors, and sizes. The environment is altered several times per week by switching and moving around toys so that the rodents are always learning to navigate a novel environment. The effects of living in EE has been shown to result in enhanced neurogenesis, synaptogenesis, and angiogenesis [19,20,21,22], as well as increased dendritic arborization, growth factor levels [e.g., brain-derived neurotrophic factor (BDNF)], and cognition-linked gene expression [21,23,24,25,26]. These neural changes are accompanied by positive effects on behavior, including enhanced spatial and working memory [21,27,28], as well as reduced anxiety- and depression-related behaviors [29,30,31]. The benefits of EE in healthy animals led to testing the effects of EE in several mouse models of AD pathology. Generally, studies have found that EE attenuates cognitive deficits; however, pathological findings have been mixed. Some studies have found that EE exposure results in cognitive benefits and reduced AD-related pathology [32,33] while another study showed benefits via amyloid-dependent and -independent mechanisms [34]. Other studies on EE found improved cognition in the absence of effects on pathology [35]. On the other hand, some studies have shown that EE improves cognitive performance while actually exacerbating amyloid pathology [36,37], perhaps by enhancing brain/cognitive reserve [38] or other mechanisms, such as reduced neuroinflammation.

While the full EE treatment, with its multiple components (novel objects, social stimulation, and physical activity), is a powerful manipulation, the contributions of each of the individual EE factors on AD pathology has been the subject of only limited study [39,40,41,42,43]. Further, no studies on EE have been performed on CAA. The present experiment used the Tg-SwDI mouse model of CAA to examine the contribution of these EE factors, alone and in combination, to differential outcomes in physiology, pathology, and behavior associated with this condition. From 4–8 months of age, mice were single housed in standard conditions, single housed with cognitive enrichment, group housed (social enrichment), or housed in a standard enriched environment and tested for physiological, cognitive/behavioral, and neuropathological outcomes. We found that individual facets of enrichment can affect this model of CAA, though social housing and combined EE conditions are generally the most effective at producing changes in physiology, cognition/behavior, and neuropathology.

## 2. Results

**Figure 1:**
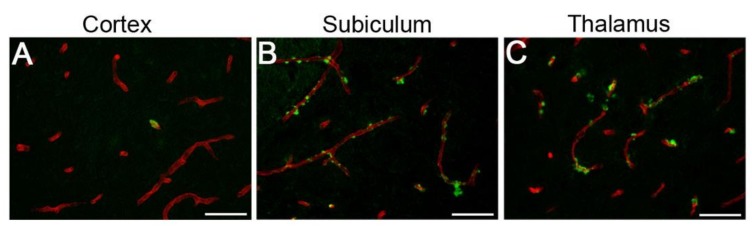
Cerebral microvascular amyloid accumulation in Tg-SwDI mice. Representative brain sections from 9-month-old Tg-SwDI mice stained with thioflavin S to identify fibrillar microvascular amyloid (green) and immunolabeled with an antibody to collagen IV to identify cerebral blood vessels (red). Microvascular amyloid deposits are observed in the cortex (**A**), subiculum (**B**), and thalamus (**C**). Scale bars = 50 µm.

Tg-SwDI mice are a transgenic mouse model of cerebral microvascular CAA type-1. Tg-SwDI mice begin to develop cerebral microvascular amyloid deposition starting at ~3–4 months of age. By 9 months of age, Tg-SwDI mice exhibit some CAA in the cortex, with more extensive deposition in the subiculum and thalamic regions (Figure 1).

**Figure 2:**
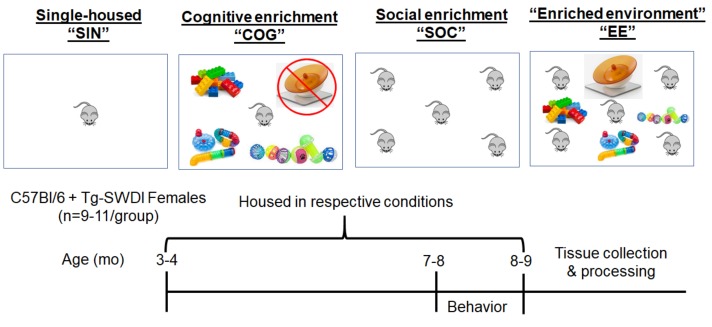
Overview of housing conditions and timeline of the experiment. At ~3–4 months of age, C57Bl/6 and Tg-SwDI female mice were randomly assigned to one of four housing conditions: SIN (single housed), COG (single housed with cognitively stimulating toys switched out twice weekly), SOC (group housed), and EE (group housed mice with toys and running wheel). Following 4 months in the respective housing conditions, mice underwent a battery of behavior tests, and tissue was collected (end age = ~8–9 months). Within housing conditions, access to a running wheel is indicated by the presence of an orange exercise saucer. Cognitive enrichment is indicated by the presence of multi-colored blocks, balls, and tunnels.

A timeline of the experiment is shown in Figure 2. Briefly, wild-type (WT) and Tg-SwDI female mice were single-housed (SIN), single-housed with cognitive enrichment (COG), socially housed (SOC), or housed in a combined enriched environment condition consisting of cognitive, social, and physical (exercise wheel) enrichment (EE). Housing interventions started when mice were ~3–4 months of age (when cerebral microvascular amyloid begins to appear) and lasted for 4 months (an age when microvascular amyloid is extensive; see Figure 1), after which several physiological, cognitive/behavioral, and neuropathological measures were assessed.

### 2.1. Physiological Measures

#### 2.1.1. Food Intake and Body Weight

**Figure 3:**
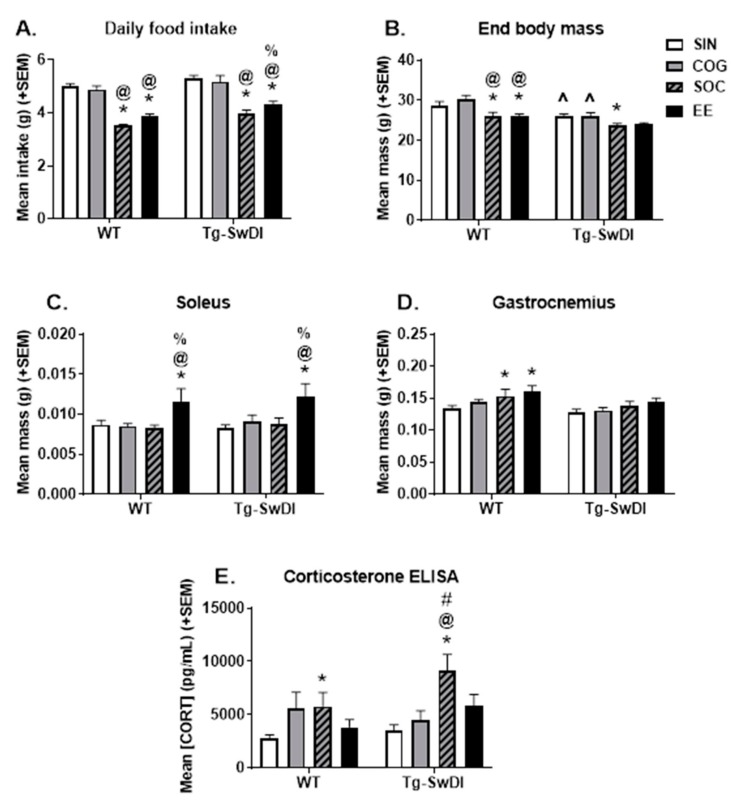
Physiological measures. (**A**) Mean daily food intake over the course of the 4-month intervention period. Generally, Tg-SwDI mice ate more than WT mice, while SOC and EE housing attenuated food intake in WT and Tg-SwDI mice. (**B**) Body weight at the end of the experiment. Generally, the Tg-SwDI mice weighed less than the WT mice. SOC (both WT and Tg-SwDI), and EE (WT only) reduced body weight. (**C**) Soleus mass was increased by EE housing in both WT and Tg-SwDI mice. (**D**) Overall, Tg-SwDI mice tended to have a smaller gastrocnemius. There were trends of SOC and EE mice of both genotypes having a larger gastrocnemius, but this was only significant in WT mice. (**E**) Corticosterone levels measured by ELISA. * *p* < 0.05 vs. SIN of the same genotype, @ *p* < 0.05 vs. COG of the same genotype, % *p* < 0.05 vs. SOC of the same genotype, # *p* < 0.05 vs. EE of the same genotype, ^ *p* < 0.05 vs. WT in the same housing condition.

To assess if the different housing conditions affected the average daily food intake and body weight, these measurements were performed at the end of the experiment and are shown in Figure 3A,B, respectively. Tg-SwDI mice weighed less (main effect of genotype, *p* < 0.001) despite eating slightly, but significantly, more compared to WT mice (main effect of genotype, *p* < 0.001). These trends were apparent, but not always statistically significant, across housing conditions. There was also a main effect of housing condition (*p* < 0.001), such that SIN and COG mice ate similar amounts and more than SOC and EE groups, while EE mice also ate more than SOC mice (*p* < 0.01 for all).

#### 2.1.2. Muscle Mass

To determine the effect of the housing conditions on muscle mass, the soleus (Figure 3C) and gastrocnemius (Figure 3D) muscles were dissected out and weighed upon euthanasia. There was no difference in the soleus mass between the two genotypes. There was a main effect of housing condition (*p* < 0.001), with EE having a larger soleus compared to all other groups (*p* < 0.001 for all), and this was consistent across both genotypes (*p* < 0.05 for all). These trends of EE mice having an increased muscle mass were also observed when normalized to the body weight (*p* < 0.01 for all).

There was a main effect of genotype (*p* = 0.009), such that Tg-SwDI mice had a smaller gastrocnemius muscle compared to WT mice, though pairwise comparisons within each housing condition did not reach significance. There was also a main effect of housing (*p* = 0.009), with EE mice having a larger gastrocnemius than SIN and COG mice, and SOC mice having a larger gastrocnemius than SIN mice (*p* < 0.05 for all). These trends were consistent but not always significant within individual genotypes. In WT mice, SOC and EE had a larger gastrocnemius than SIN mice (*p* < 0.05 for both), while in T-SwDI mice, the difference between EE and SIN mice only approached significance (*p* = 0.066). When muscle mass was normalized to body weight, both EE- and SOC-housed mice had a larger gastrocnemius compared to the SIN and COG groups (*p* < 0.01 for all except Tg-SwDI SOC vs. COG *p* = 0.089). These findings indicate that the EE group had a higher muscle mass, regardless of genotype, likely attributed to the exposure to the running wheel and increased exercise.

#### 2.1.3. Corticosterone ELISA

To assess the effect of the housing condition on stress levels in the mice, serum was collected at the time of euthanasia and corticosterone levels were measured by ELISA (Figure 3E). Neither the main effect of genotype, nor the genotype x housing interaction, were significant for serum corticosterone levels. The main effect of housing was significant (*p* = 0.0012), such that enrichment conditions tended to increase corticosterone levels though this did not always reach significance (COG *p* = 0.0748, SOC *p* < 0.0001, EE *p* = 0.1146). Within WT mice, COG (*p* = 0.0575) and SOC (*p* = 0.0432) housing increased corticosterone levels compared to SIN mice. Within Tg-SwDI mice, SOC mice had higher corticosterone levels compared to all other housing conditions (*p* < 0.05 for all).

### 2.2. Behavioral Tasks of Motor Function and Temperament

#### 2.2.1. Rotarod

**Figure 4:**
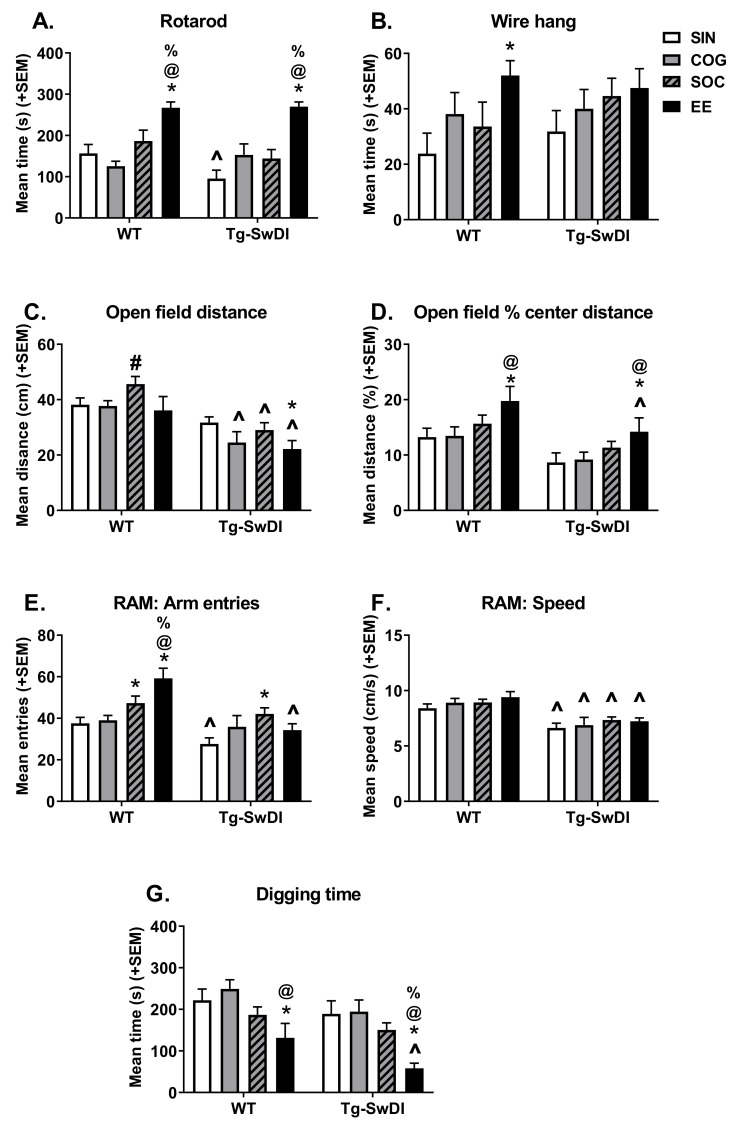
Activity and temperament measures. (**A**) Rotarod performance measured by time spent on rod (average of best two of three trials), indicative of balance and motor coordination. Under SIN conditions, Tg-SwDI mice were impaired in this task, while EE housing improved performance in both WT and Tg-SwDI mice. (**B**) Wire hang performance measured by time spent hanging onto a wire, indicative of forelimb strength. EE housing improved performance, but this was only significant in WT mice. (**C**) General activity levels, as measured by the distance traveled in the open field. SOC housing increased activity in WT mice. Overall, Tg-SwDI mice were hypoactive and this was exacerbated in EE mice. (**D**) Anxiety-like behavior, as measured by the percent of distance traveled in the center of the open field arena. Overall, Tg-SwDI mice exhibited increased anxiety-like behavior (less center activity), while anxiety was reduced by EE housing in both WT and Tg-SwDI mice. E and F) Exploratory behavior, as measured by the number of arm entries (**E**) and speed of travel (**F**) in the radial arm maze task. Generally, Tg-SwDI mice exhibited lower levels of exploration, and the number of arm entries was increased in SOC mice. In WT mice, SOC and EE housing increased the number of arm entries as well. (**G**) Time spent digging in a marble-burying task. Generally, Tg-SwDI mice exhibited lower levels of digging behavior, while EE housing attenuated digging behavior in both WT and Tg-SwDI mice. * *p* < 0.05 vs. SIN of the same genotype, @ *p* < 0.05 vs. COG of the same genotype, % *p* < 0.05 vs. SOC of the same genotype, # *p* < 0.05 vs. EE of the same genotype, ^ *p* < 0.05 vs. WT in the same housing condition.

Rotarod was performed to assess motor coordination and balance (Figure 4A). In SIN conditions, Tg-SwDI mice were impaired on the rotarod compared to WT mice (*p* = 0.028). However, in all other housing conditions, both genotypes performed similarly. There was an overall main effect of housing (*p* < 0.001), with EE mice performing better than mice in all other housing conditions (*p* < 0.001 for all); these trends were significant within both WT and Tg-SwDI mice (*p* < 0.05 for all). In Tg-SwDI mice, there were also trends of COG (*p* = 0.050) and SOC (*p* = 0.085) conditions improving performance.

#### 2.2.2. Wire Hang

Wire hang was performed to assess forelimb strength (Figure 4B). Tg-SwDI mice did not perform differently from WT mice under any housing conditions. There was an overall main effect of housing (*p* = 0.041), with EE mice performing better than mice housed in SIN conditions (*p* = 0.005); however, pairwise comparisons revealed that this was significant in WT mice only (*p* = 0.012).

#### 2.2.3. Open Field

Open field was performed to assess general activity levels (distance traveled; Figure 4C) and anxiety-like behavior (center activity; Figure 4D). There was a main effect of genotype (*p* < 0.001), such that Tg-SwDI mice were less active in the open field compared to WT mice. This trend was consistent in all housing conditions (*p* < 0.05 for all but SIN *p* = 0.10). The main effect of housing was also significant (*p* = 0.036), though trends were not consistent across genotypes. In WT, SOC mice were more active than mice in all other housing conditions (SIN *p* = 0.074, COG *p* = 0.061, EE *p* = 0.033). In Tg-SwDI, enriched housing conditions did not rescue activity deficits; in fact, Tg-SwDI EE mice were less active than mice housed in the SIN condition (*p* = 0.023).

There was a main effect of genotype (*p* < 0.001), such that Tg-SwDI mice exhibited less center activity compared to WT mice. This trend was consistent in all housing conditions, though did not always reach statistical significance (SIN *p* = 0.065, COG *p* = 0.095, SOC *p* = 0.086, EE *p* = 0.042). There was also a significant main effect of housing (*p* = 0.005), such that EE mice exhibited greater center activity compared to all other housing conditions (*p* < 0.05 for all but SOC *p* = 0.067). When analyzed within individual genotypes, in both WT and Tg-SwDI mice, EE housing increased center activity compared to SIN and COG mice (*p* < 0.05 for all) but not SOC mice.

#### 2.2.4. Unreinforced Radial Arm Maze

The unreinforced radial arm was performed to assess exploratory behavior in a relatively complex environment by measuring the number of arm entries (Figure 4E) and speed (Figure 4F). There was a main effect of genotype for the radial arm maze (RAM) entries (*p* = 0.0151), with Tg-SwDI mice making fewer arm entries compared to WT mice. When analyzed within housing conditions, this was significant for SIN (*p* = 0.0365) and EE (*p* < 0.0001) mice. The main effect of housing was also significant (*p* = 0.0003), such that SOC and EE mice made a greater number of arm entries compared to SIN and COG mice (*p* < 0.05 for all). The genotype x housing interaction was also significant (*p* = 0.0151). In WT mice, there was an apparent stepwise effect of enrichment (EE > SOC > COG = SIN; *p* < 0.05 for all except SOC vs. COG *p* = 0.0931). Within Tg-SwDI mice, only the SOC condition significantly increased arm entries compared to SIN mice (*p* = 0.0034).

There was a main effect of genotype for speed in the radial arm maze (*p* < 0.0001), with Tg-SwDI mice moving more slowly than WT mice, and this trend was consistent across housing conditions. The main effect of housing and the genotype x housing interactions were not significant.

#### 2.2.5. Digging

A marble-burying task was used to assess digging, a species-typical behavior (Figure 4G). There was a main effect of genotype (*p* = 0.008), such that Tg-SwDI mice exhibited less digging compared to WT mice; however, within the individual housing conditions, this only reached significance for EE mice (*p* = 0.027). There was also a significant main effect of housing (*p* < 0.001), with SOC and EE mice digging less than SIN and COG mice (*p* < 0.05 for all except SIN vs. SOC *p* = 0.072). Additionally, EE mice dug less than SOC mice (*p* = 0.006). These trends were consistent across genotypes, though not all were statistically significant. In both WT and Tg-SwDI mice, EE mice dug less than SIN and COG mice (*p* < 0.05 for all). Only in Tg-SwDI mice did EE dig significantly less than SOC mice (*p* = 0.005).

### 2.3. Cognitive Behavioral Performance

#### 2.3.1. Novel Object Recognition

**Figure 5:**
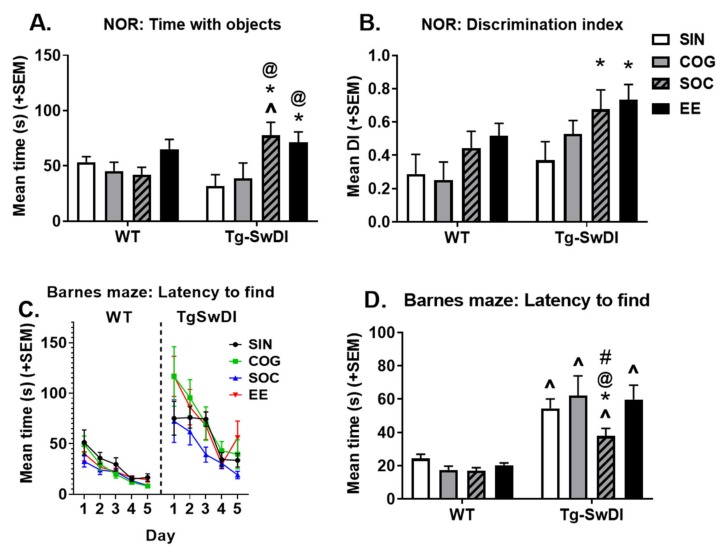
Cognitive testing. In Tg-SwDI mice, SOC and EE housing increased the amount of time spent with objects (**A**), and the discrimination index (**B**) in the novel object recognition (NOR) task. (**C**) Barnes maze performance, as measured by the latency to find the escape hole over time. (**D**) Barnes maze performance, as measured by the average latency to find the escape hole on days 2–5. Tg-SwDI mice took longer to find the escape hole compared to WT mice in all housing conditions. In Tg-SwDI mice, SOC housing was associated with improved performance (shorter latency to find the escape hole). * *p* < 0.05 vs. SIN of the same genotype, @ *p* < 0.05 vs. COG of the same genotype, % *p* < 0.05 vs. SOC of the same genotype, # *p* < 0.05 vs. EE of the same genotype, ^ *p* < 0.05 vs. WT in the same housing condition.

A novel object recognition test was performed to assess non-spatial memory. The total time exploring both objects (Figure 5A) and the discrimination index for exploration of the novel object (Figure 5B) were measured. The main effect of housing condition (*p* = 0.013) and the genotype x housing interaction (*p* = 0.020) were significant. In the SIN condition, Tg-SwDI mice explored objects less than WT mice, though this only approached significance (*p* = 0.086), while this was reversed in the SOC condition (Tg-SwDI > WT, *p* = 0.009). In WT mice, pairwise comparisons revealed no significant differences between groups; however, in Tg-SwDI mice, SOC and EE mice explored objects more than SIN and COG mice (*p* < 0.05 for all).

The main effect of genotype was significant for the discrimination index in the novel object recognition (NOR) test, with Tg-SwDI mice outperforming WT mice (*p* = 0.009). Although pairwise comparisons revealed this was not statistically significant within any of the individual housing conditions, this genotype effect seemed to be driven more by enriched conditions and less so by the SIN condition in which discrimination indices were more comparable. Additionally, the main effect of housing was significant (*p* = 0.019), with EE and SOC groups displaying increased performance. In WT mice, pairwise comparisons revealed no statistically significant differences between groups; however, in Tg-SwDI mice, both SOC and EE groups outperformed SIN mice (*p* < 0.05 for both).

#### 2.3.2. Barnes Maze

The Barnes maze task was run over a period of five days, with two trials per day. Spatial learning and memory were assessed by the measure of latency to find the escape hole over the course of the five days (Figure 5C) and the average latency to find on days 2–5 (Figure 5D). Negative slopes of latency to find over the course of the five days indicated that all groups were able to learn the task over time. There was a striking main effect of genotype (*p* < 0.001), with Tg-SwDI mice finding the escape box more slowly than WT mice, regardless of the housing condition. Within WT mice, enriched groups generally outperformed SIN mice, but these comparisons were not significant. Within Tg-SwDI mice, SOC mice found the escape hole more quickly than mice in all other housing conditions (*p* < 0.05 for all).

### 2.4. Pathology

#### 2.4.1. ELISAs for Aβ Species

**Figure 6:**
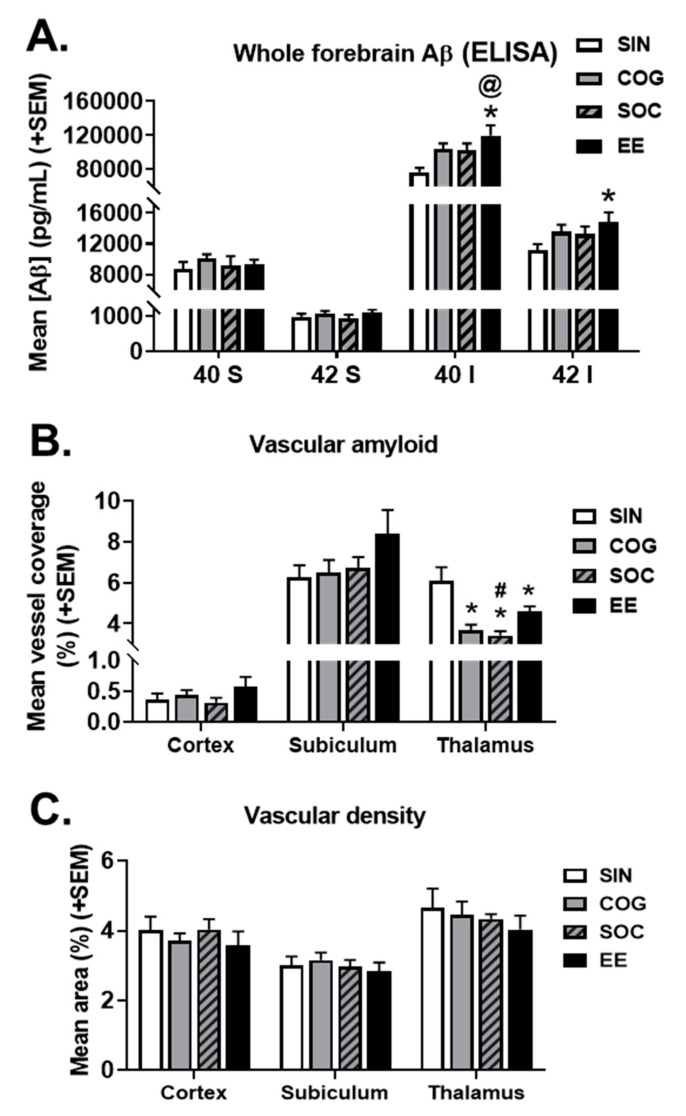
Effects of the housing condition on the accumulation of Aβ in Tg-SwDI mice. (**A**) Tg-SwDI mice housed in EE conditions had increased insoluble Aβ40 and Aβ42) levels, as measured by ELISA of whole forebrain homogenates. (**B**) Tg-SwDI mice in all enrichment conditions had less vascular amyloid in the thalamus compared to single-housed mice. (**C**) The housing condition did not affect the cerebral vascular density in any of the three brain regions measured. * *p* < 0.05 vs. SIN of the same genotype, @ *p* < 0.05 vs. COG of the same genotype, # *p* < 0.05 vs. EE of the same genotype.

ELISAs were performed on whole forebrain homogenates to assess the effect of the housing condition on the levels of Aβ species (Figure 6A). There were no effects of the housing condition on soluble Aβ species (40 or 42); however, thee housing condition did affect the forebrain levels of insoluble Aβ40 (*p* = 0.010) and Aβ42 (*p* = 0.064). Surprisingly, pairwise comparisons revealed that all enrichment conditions increased insoluble Aβ40 levels compared to SIN mice (*p* < 0.05 for all but SOC *p* = 0.053). Similar trends were seen for insoluble Aβ42; however, only EE housing led to a statistically significant increase (*p* = 0.009). Since WT mice do not accumulate microvascular amyloid, they were not included in these measures.

#### 2.4.2. Microvascular Amyloid Deposition

Since there were effects of the housing condition on the accumulation of insoluble Aβ, particularly insoluble Aβ40, we measured the extent of microvascular amyloid deposition in different brain regions (Figure 6B). One-way ANOVAs found no effect of housing on microvascular amyloid deposition in the cortex or subiculum; however, there was a significant effect in the thalamus (*p* < 0.001), with all enrichment groups having reduced vascular amyloid compared to SIN mice (*p* < 0.05 for all). Additionally, SOC mice had less vascular amyloid compared to EE mice (*p* = 0.045).

#### 2.4.3. Vascular Density

Since the accumulation of insoluble Aβ tended to increase, whereas the microvascular CAA load was either unchanged or in the case of the thalamus was reduced, we next determined if the EE conditions impacted the cerebral vascular density in Tg-SwDI mice. Cerebral vascular density, however, was not affected by housing condition in any of the regions measured (cortex, subiculum, and thalamus) (Figure 6C).

## 3. Discussion

Lifestyle factors, such as exercise and participating in cognitively and socially stimulating activities, promote healthy aging and prevent the risk of dementia in elderly populations [11,12,13,14,15,16,17,18]. These findings are supported by studies in healthy mice [19,20,21,22,23,24,25,26,27,28,29,30,31] and rodent models of AD [32,33,34,35,36,37], often combining these three factors in the enriched environment (EE) paradigm. Few studies have aimed to directly compare and tease apart the contribution of individual and combined EE factors in AD mouse models, and those that did utilized models that primarily develop parenchymal amyloid [39,40]. This study, for the first time, determined the protective effects of environmental enrichment (EE) in the Tg-SwDI mouse model of CAA, a condition characterized by the accumulation of cerebral vascular amyloid found to be common in aging and seen in the vast majority of AD cases [1]. We also disassembled the EE paradigm to determine the unique contributions of its cognitively and socially stimulating components.

Mice housed in the cognitively enriched condition (COG; single housed with toys and tunnels switched out 2×/week to provide novelty) exhibited few cognitive/behavioral benefits compared to single-housed mice without such added enrichment. Tg-SwDI mice housed in the COG condition did display improved performance on some tasks of motor function (rotarod) and exploratory behavior (radial arm maze), in addition to reduced cerebral microvascular amyloid pathology in the thalamus. Mood- and cognition-related behavior was unaffected by cognitive stimulation, in line with our previous findings in Tg-SwDI and 5xFAD mice. In these strains, we provided cognitive enrichment using a progressive cognitive stimulation paradigm meant to model commercialized brain training games in humans, which included 4 months of progressively difficult domain-specific operant tasks [44]. Results from this previous study supported clinical findings that while brain training games enhance performance on the specific task being trained, improvements do not generalize to other cognitive domains to improve global function, promote brain health, or prevent cognitive decline [45,46,47,48].

On the other hand, the current study provides evidence that socially housed mice, and those housed in a completely enriched environment, displayed the greatest changes in physiological and cognitive/behavioral outcomes. These mice, in general, exhibited a similarly reduced body weight and food intake, and improved performance on the novel object recognition task. As expected, the EE condition produced greater changes for some outcomes, such as muscle mass, tests of motor function and activity levels, and anxiety-like behavior, likely due to the addition of access to a running wheel to engage in voluntary aerobic exercise. Similarly, we previously reported the dose-dependent effects of aerobic exercise (voluntary wheel running) in WT mice and the Tg-SwDI model, with even relatively small amounts of exercise exerting notable benefits to physiology, motor function, anxiety, and cognition [49,50]. It should be noted that social and EE housing also reduced food intake and body weight in both WT and Tg-SwDI mice. Caloric restriction has been shown to improve memory and reduce dementia risk in humans, and increase cognitive function in animal models of healthy aging and AD [51,52,53,54,55,56]. These cognitive behavioral benefits are associated with decreased inflammation, oxidative stress, and AD pathology, and increased insulin sensitivity, autophagy, and neurogenesis [54,57,58,59]. In the current study, the reduced caloric intake displayed by SOC and EE mice of both genotypes fall approximately within the range of 20–40% typically used in rodent studies (WT SOC: −29%, WT EE: −23%, Tg-SwDI SOC: 25%, Tg-SwDI EE: 18%). Additionally, caloric deficits were likely exacerbated by increased energy output, particularly in EE mice with access to a running wheel. The likelihood of increased physical activity and therefore caloric expenditure by these groups is supported by their increased muscle mass. Therefore, one possibility is that enriched housing conditions exert behavioral benefits indirectly by affecting caloric intake and/or metabolic outcomes.

Interestingly, though thalamic vascular amyloid was specifically reduced by all forms of enriched housing, the levels of insoluble Aβ as detected by ELISA were actually somewhat increased in Tg-SwDI mice housed in the EE condition. Although the nature of this increase is unclear, this is in agreement with early studies by Jankowsky et al. [36,37], which found that six months of housing in an enriched enrichment also increased Aβ levels in APPswe and APPswe/PS1dE9, despite these mice showing enhanced performance in spatial memory tasks (Morris water maze and radial water maze) [36,37]. While there were trends of COG and SOC mice also having increased insoluble Aβ in our study, these effects were not significant. Therefore, these types of enrichment stimulation appear to have additive incremental effects on insoluble Aβ levels. As in previous studies, we found that these increased levels of insoluble Aβ occurred in the presence of behavioral improvement and were therefore not detrimental to functioning, in agreement with previous findings that insoluble Aβ exhibits low toxicity [60,61,62]. Previously, we showed that exercise alone was also capable of dose-dependently increasing insoluble Aβ levels, despite some behavioral improvements and attenuated neuroinflammation [49]. Taken together, these findings suggest that enrichment factors increase the production/aggregation or reduce the degradation/clearance of Aβ, though vascular amyloid was specifically reduced in some brain regions. One possible explanation for the former is that enriched housing may increase neuronal activity, which could enhance Aβ production and deposition [63,64]. The effects of environmental enrichment on Aβ production and clearance in Tg-SwDI mice should be further assessed. Moreover, enrichment could affect other measures rather than insoluble Aβ to influence behavior and cognition, such as reducing neuroinflammation and increasing hippocampal growth factors and neurogenesis [65,66,67]. Investigation of these other possible mechanisms could not only offer an alternative explanation to Aβ burden in Tg-SwDI animals but also explain enrichment effects within WT mice.

This study represents one of the first to disentangle the contributions of EE factors in a mouse model of CAA. In a previous study, APP-23 mice were housed in standard or enriched conditions, or in cages with access to a running wheel. Enriched mice had improved performance on the Morris water maze despite an unchanged amyloid burden, though they did exhibit increased levels of BDNF and hippocampal neurogenesis. In contrast, exercise had no effect on cognitive performance or neurogenesis; however, there was a decrease in growth factors in the cortex and hippocampus [39]. In another study, APPsw/APP + PS1 mice were grouped into environments that layered on these factors (impoverished, social, social + physical, complete enrichment) from 1.5–9 months of age. Only the completely enriched environment was capable of protecting against cognitive decline, reducing Aβ deposition, and increasing synaptic reactivity in the hippocampus, while no differences between groups in corticosterone or inflammatory cytokines were observed [40]. Recent clinical findings also provide evidence that adopting multiple healthy lifestyle factors exerts a benefit on the body and brain. The Finnish Geriatric Intervention Study to Prevent Cognitive Impairment and Disability (FINGER) study was a randomized controlled trial to assess a two-year multidomain intervention (diet, exercise, cognitive training, vascular risk monitoring), finding it effective at preventing cognitive decline in an at-risk elderly population [68]. Recently reported at the 2019 Alzheimer’s Association International Conference (AAIC), a study using the data of over 2000 people from the Chicago Health and Aging Project and the Rush Memory and Aging Project determined that aging adults that exhibited four to five healthy lifestyle factors (diet, exercise, and cognitive stimulation, in addition to smoking and alcohol use) had the greatest reduction in risk for AD (~60%). Additionally, regardless of the number of current healthy lifestyle factors exhibited, adopting just one more reduced the risk by 22% [69]. Other data presented at the 2019 AAIC suggest that a healthy lifestyle can even counteract genetic risk for dementia, boasting a 32% reduction [70]. Our findings, for the first time, show that EE can be beneficial by specifically targeting CAA, and add to a growing literature supporting that the adoption of a healthy lifestyle is key to healthy aging, and specifically brain health, and for reducing the risk of cognitive decline and dementia in later life.

## 4. Materials and Methods

### 4.1. Animals

Female C57BL/6 (wild type; WT) and Tg-SwDI mice were used in this experiment. The Tg-SwDI mouse is a model of CAA, in which fibrillar Aβ accumulates primarily in the cerebral microvasculature, apparently, in part, due to insufficient clearance of the protein across the blood–brain barrier [71]. These mice are on a C57BL/6 background and express low levels of the human amyloid precursor protein (APP) gene, containing the Swedish K670N/M671L, Dutch E693Q and Iowa D694N mutations, under the control of the mouse *Thy1* promoter [72]. CAA pathology is accompanied by vascular degeneration and marked gliosis [73], as well as impaired Barnes maze performance not attributable to deficits in mobility, strength, or coordination [74].

Mice were housed in a controlled room (22 ± 2 °C and 40–60% humidity) with a 12-h reverse light-dark cycle (lights off 0800 h). Mice were habituated for one week prior to the beginning of the experiment, and then split into experimental treatment groups at 3–4 months of age. Purina Lab Diet rat chow was available ad libitum, and body weight and food intake were recorded weekly throughout the entire experiment. All experiments were conducted in conformity with the National Academy of Sciences Guide for Care and Use of Laboratory Animals and approved by the Stony Brook University Institutional Animal Care and Use Committee. (Project identification code: 2013-0788; date: 17 November 2014)

### 4.2. Housing Conditions

Mice of each genotype were split into experimental housing conditions: WT single-housed (WT SIN; *n* = 11), WT cognitively enriched (WT COG; *n* = 10), WT socially enriched (WT SOC; *n* = 10), WT fully enriched (WT EE; *n* = 8), Tg-SwDI SIN (*n* = 11), Tg-SwDI COG (*n* = 9), Tg-SwDI SOC (*n* = 10), and Tg-SwDI EE (*n* = 10). SIN mice were housed one per cage. COG mice were housed one per cage, with toys (colored blocks, balls, and tunnels) that were changed out twice weekly. SOC mice were group housed (4–6 mice/cage). EE mice were group housed (4–6 mice/cage) in a cage equipped with an exercise wheel and toys (colored blocks, balls, and tunnels) that were changed out twice weekly. Mice remained in these housing conditions for the duration of the experiment. An illustration of housing conditions and timeline of the experiment can be seen in Figure 1.

### 4.3. Cognitive/Behavioral Assessments

Following four months of housing in the respective conditions, all mice underwent a battery of behavioral testing, including the rotarod, wire hang, open field, unreinforced radial arm maze, marble burying, novel object recognition, and Barnes maze. Mice remained in their housing conditions throughout the duration of testing. All behavior testing, except for rotarod, was recorded and analyzed using ANY-maze tracking software.

#### 4.3.1. Rotarod

Rotarod was performed to assess balance, strength, and motor coordination. The apparatus (model ENV-575M; MED Associates Inc.) is composed of a 30-cm-long rod that is divided into five equally sized 6-cm sections. The mice were placed on the rotarod, which spun at an increasing speed of up to 40 revolutions per minute over a five-minute period. The time on the rod until the mouse fell (maximum time of five minutes) was recorded. The mice were tested three times, with a minimum five-minute inter-trial interval, and the average of the last two trials was used for the analysis.

#### 4.3.2. Wire Hang

The wire hang consisted of a single trial to assess muscle strength/endurance using an apparatus consisting of a 43-cm-long wire stretched between two wooden poles, 48 cm high above the base. Foam padding was placed on the platform to cushion a fall. At the beginning of the trial, the mouse’s two front paws were placed on the wire and the mouse was allowed to hang until it fell or for a maximum of one minute. The latency to fall was recorded (maximum one minute).

#### 4.3.3. Open Field

The mice were placed in a square 60 cm x 60 cm open field arena for 10 min. Behavior was recorded using ANY-maze software. General locomotor behavior and motor function was assessed using the measure of the distance traveled in the open field. Since mice are agoraphobic, anxiety-like behavior was assessed by the time spent in the center of the open field [75]. Measures of general activity and agoraphobia-related anxiety are also useful in the interpretation of any differences in performance observed in other cognitive/behavioral tasks.

#### 4.3.4. Unreinforced Radial Arm Maze

The mice were placed in a radial arm maze with eight arms for five minutes. The number of arm entries was assessed as a measure of exploratory behavior in a novel and relatively complex environment (compared to the open field).

#### 4.3.5. Marble Burying

The protocol for marble burying was adapted from a well-defined protocol [76]. The mice were place in a rat-sized tub cage filled with 5 cm of corn cob bedding for five minutes with 20 marbles in a 5 x 4 array, during which time the time spent digging was recorded. Digging was defined as coordinated movements of the fore or hind limbs that displaced the bedding.

#### 4.3.6. Novel Object Recognition

A novel object recognition task was performed to assess non-spatial learning and memory (Figure 5). This task consisted of two trials, each lasting 5 min, with an inter-trial interval of 15 min. In the first trial, two of the same objects were placed in the open field arena. In the second trial, one object was replaced by a novel object, while the other object remained the same and in the same location. The time spent exploring both objects was counted, and novel object recognition was assessed by calculating the discrimination index (DI). DI = (time with novel object—time with familiar object)/(time with novel object—time with familiar object).

#### 4.3.7. Barnes Circular Maze

The Barnes maze was originally developed to test learning and memory in rats. We used an adaptation of this maze, a circular wooden platform, 91 cm in diameter, elevated 75 cm off the ground. The platform has eight equally spaced escape holes along the periphery that are 24.5 cm apart. Under each hole, a shelf securely held an escape box, measuring 10 cm x 8.5 cm x 4 cm. There were visible distal cues placed around the room, which remained constant throughout the duration of testing. Testing were performed on five consecutive days, with two trials per day separated by a 15-min inter-trial interval. Mice were placed onto the center of the maze at the beginning of each trial, then allowed to explore until the escape box was found and entered, or a maximum of five minutes. If the escape box was entered, the mouse remained there for one minute before being transferred back to its home cage. If the escape box was not entered within five minutes, the mouse was placed in the escape box and left there for one minute. During each trial, the following measures were recorded: Latency to find (amount of time taken to find the escape box), latency to enter (amount of time taken to enter the escape box), and errors (number of nose pokes into a hole that did not contain the escape box before finding the escape box).

### 4.4. Physiological Measures

#### 4.4.1. Blood, Organ, and Muscle Collection

Following the completion of behavioral testing, mice were euthanized under deep anesthesia with 2.5% avertin. Cardiac puncture was performed to collect blood, which was allowed to clot at room temperature for 30 min, spun at 2000× g for 10 min, and serum was collected and stored at –80 °C until use in the assays. Following blood collection, mice were perfused with saline. Muscles were collected to assess exercise-induced differences in muscle mass. After weighing, brains were bisected along the midline and prepared for subsequent pathological analyses. One hemisphere was placed in 70% ethanol and subsequently paraffin embedded for immunohistochemistry, while the other was flash frozen in liquid nitrogen for ELISAs and quantitative polymerase chain reaction.

#### 4.4.2. Enzyme Linked Immunosorbent Assay (ELISA) For Serum Corticosterone

Serum samples were analyzed using a commercially available ELISA for corticosterone according to the manufacturer’s instructions (Cayman Chemical). Absorbance was recorded using a plate reader (Spectramax).

### 4.5. Pathological Aβ Measures

#### 4.5.1. Enzyme-Linked Immunosorbent Assay (ELISA) For Aβ Species

ELISAs were performed to quantify whole brain levels of membrane, soluble, and insoluble forms of Aβ40 and Aβ42. Brain hemispheres that were flash frozen were pulverized and separated into three aliquots. A soluble fraction was obtained by homogenized tissue with 10 µL/mg TBS using 0.5 mm glass beads and a bullet blender. Aliquots were centrifuged at 1600× g at 4 °C for 20 min. The supernatant was removed, which was the soluble fraction. The remaining pellet was resuspended in 5 M guanidine-HCl at pH 8.0, and rotated at room temperature for 3 h. Samples were centrifuged as above, and the supernatant was removed, which was the insoluble fraction. For each fraction, a sandwich ELISA for Aβ was performed using antibody reagents generously provided by Eli Lilly (Indianapolis, IN). Well plates were coated with 1 µL/well of Aβ40-specific antibody M2G3 or Aβ42-specific antibody M21F12. Plates were blocked and shaken overnight at 4 °C. Aβ species were detected using biotinylated-m3DG, followed by streptavidin-HRP (Amdex RPN4401V). Plates were developed using SureBlue (KPL) and plates were read with a plate reader (Spectramax).

#### 4.5.2. Immunostaining and Analysis

Immunohistochemical analyses were performed as described in Xu et al. (2007) on Tg-SwDI brain tissue (*n* = 6–7/group). Brain hemispheres embedded in paraffin were sectioned at a 10-µM thickness and mounted on glass slides. Paraffin was removed from sections by immersion in xylene (3 × 5 min) and rehydrated in decreasing concentrations of ethanol (100%, 95%, 70%, 50%, 0% at 5 min each). Slides were dipped in PBS for 5 min, followed by a 5 min incubation with proteinase K (1:1000 in PBS) for antigen retrieval, then dipped in distilled water 5x 1min. Buffer consisting of 0.3% Triton X-100 was used to block sections for 30 min, which were then incubated with primary antibody (1:100 collagen type IV for blood vessels) in a 1:10 0.1% Triton X-100 blocking buffer solution overnight at room temperature. The next day, slides were washed 3 × 5 min with distilled water, then incubated for 2 h with secondary antibody (1:1000 594 anti-Rabbit) in a 1:10 0.1% Triton X-100 blocking buffer solution. Slides were rinsed with distilled water. Slides were then stained for Thioflavin-S (0.0125% Thioflavin-S in 50% EtOH/PBS) by incubating for 15 min at room temperature. Slides were rinsed 3× with distilled water then 2× with 70% EtOH and then washed for 5 min in distilled water. Anti-fade reagent in glycerol/PBS was added to each slide and coverslipped, then sealed with mounting media. Sections were imaged using an Olympus BX60 microscope with an attached Olympus Dp72 camera. Images from the cortex, subiculum, and thalamus were collected from each section at the 40× magnification. Using NIH ImageJ software, an appropriate threshold was set for each stain and the percent area occupied with positive stain was quantified. Fibrillar vascular amyloid deposition were assessed in the cortex, subiculum, and thalamus. These regions were chosen as they have been previously shown to accumulate cerebral microvascular amyloid and are involved in several of the behavioral tasks used in the current study. Fibrillar amyloid deposition was calculated by measuring the percentage of positive staining for thioflavin-S. Vascular amyloid deposition (percentage of blood vessel coverage with fibrillar amyloid) was calculated by [(ThioflavinS+ stain/Collagen IV+ stain) * 100]. Fibrillar vascular amyloid was only assessed in Tg-SwDI mice, as WT mice do not show any accumulation in brain. Additionally, the effects of exercise on the vascular density were assessed as the area of labeling for collagen IV in the cortex, subiculum, and thalamus.

### 4.6. Statistical Analyses

Two-way ANOVAs (factors: Genotype and housing) were performed to determine differences between groups for organ and muscle mass, serum corticosterone concentration, and behavioral and pathological measures. Three-way repeated measures ANOVAs were performed to assess differences in groups over time for food intake and body weight, as well as group differences in Barnes maze performance measures over time. Analyses were performed using Statistica and SigmaPlot/Stat, and significance was set at alpha = 0.05.

## Abbreviations

Aβ	Beta amyloid
AD	Alzheimer’s disease
APP	Amyloid precursor protein
BDNF	Brain-derived neurotrophic factor
CAA	Cerebral amyloid angiopathy
COG	Cognitively enriched treatment group
DI	Discrimination index
EE	Enriched environment
ELISA	Enzyme-linked immunosorbent assay
NOR	Novel object recognition
PBS	Phosphate buffered saline
SIN	Single-housed treatment group
SOC	Socially (group)-housed treatment group
TBS	Tris Buffered Saline
WT	Wild-type mice
